# Digital learning strategies in residency education

**DOI:** 10.1080/07853890.2024.2440630

**Published:** 2024-12-18

**Authors:** Helena Vallo Hult, Adam Abovarda, Christian Master Östlund, Paul Pålsson

**Affiliations:** a School of Business, Economics and IT, University West, Trollhättan, Sweden; b Department of Education, NU Hospital Group, Trollhättan, Sweden; c Institute of Neuroscience and Physiology, University of Gothenburg, Sahlgrenska Academy, Gothenburg, Sweden

**Keywords:** Digital learning, video conferencing, residency, medical education, physicians

## Abstract

**Background:**

New digital learning environments have transformed medical education and training, allowing students and teachers to engage in synchronous, real-time interactions and asynchronous learning activities online. Despite extensive research on the role of digital technologies in education, understanding the interplay between digital technology, work, and learning, especially in complex fields like healthcare, remains a challenge.

**Objective:**

The objective of this study is to examine resident physicians’ perceptions and experiences of using a digital learning environment as part of their specialist medical training. The paper focuses on digital learning through video conferencing (virtual lectures and seminars) and related learning technologies. It aims to understand how resident physicians perceive pedagogical opportunities and challenges in digital learning environments during their medical training and what strategies they use to address these.

**Materials and Methods:**

The methodological approach is qualitative, aiming to capture and understand participants’ experiences and views of digital learning. The empirical data gathered from open-ended responses to four course evaluation surveys and semi-structured interviews with nine physicians from a cohort of participants enrolled in two or more digital courses were analyzed through thematic analysis. The analysis revealed three main themes related to digital transformation of learning: sociotechnical, educational and administrative.

**Results:**

The results suggest that (i) sociotechnical aspects and understanding of the context in which the learning takes place contribute to enhancing digital learning for resident physicians; (ii) insights into participants’ perceptions of digital learning emphasize that interactive communication and group discussions are significant for their learning, and (iii) administrative aspects related to course design, lecture management, and instructional support are more important in digital learning environments compared to traditional teaching and learning.

**Conclusion:**

Findings from this study confirm and extend prior studies on digital learning in healthcare, contributing to a better understanding of how digital learning environments, especially virtual lectures and seminars, can be developed and integrated into residency programs and health professions education to increase their usefulness.

## Introduction

The development of digital solutions for teaching and learning has increased not only for medical schools and higher education but also for continuing education and training in the health professions [1]. New digital learning environments have enabled students and teachers to increasingly participate online and remotely in joint learning activities through synchronous, real-time interaction and communication such as video lectures and seminars and asynchronous learning through, e.g. discussion forums and recorded lectures [*cf.* 2,3]. In this paper, we use the term digital learning to describe learning and training supported by technology-mediated methods for assessment, tutoring, and instruction, including collaborative and cooperative elements [4], with a focus on synchronous, real-time interaction and learning *via* video conferencing and webinars [5]. While digital learning was widespread in higher education and professional training before the Covid-19 pandemic [5], it became the primary means for providing education when social distancing became a necessity [6,7] as it was considered the most suitable solution to tackle the situation [8–10].

Prior research has identified both benefits and limitations of digital learning, reflecting the dual nature of digital technologies as simultaneously enabling and constraining teaching and learning. On the one hand, flexibility in terms of time and space is considered a significant benefit of digital learning as it provides easy access to course material and enables the spread of knowledge to a broader range of people while remaining cost-effective in the long term [7,11]. Access to educational materials and document sharing through various methods, such as video and audio, is beneficial as it helps facilitate interactive learning activities, communication, and collaboration and allows for learning at an individual pace and avoiding stress [11]. On the other hand, research has also found that the absence of face-to-face interaction in digital learning environments may discourage engagement in the learning process, and lack of access to needed technology can limit the ability to participate and benefit from the opportunities afforded by digital technology [12,13]. Digital learning also has limitations regarding cognitive aspects, as spending many hours in video conferencing can negatively affect learners and learning due to tiredness and concentration issues, commonly referred to as Zoom fatigue [*cf.* 14].

A substantial body of literature has explored the shift to digital learning in the education of health professionals during and after the pandemic. Most of the focus has been on undergraduate students within the university and academic hospital settings, emphasizing the rapid shift to online learning and simulation [10,15–17]. These studies have contributed important knowledge about the lessons learned during the pandemic and its aftermath, and the findings have relevance to residency education and digital learning in the health professions more broadly. However, more research is still needed regarding professionals’ perspectives on digital technology in the context of workplace learning and the role of digital learning within residency education [6,10,18]. Empirical insights and experiences are therefore valuable to understanding these perspectives and can help develop strategies for how digital transformation can enhance medical education developments in undergraduate, graduate, and continuing medical education.

This paper builds on and extends previous research by the authors, where we explored the digital transformation of residency education from the perspective of the course leaders [19] and initial findings from a combined literature review and analysis of course evaluations [20]. In this paper, we take the perspective of the course participants, examining the effects of integrating digital technology into the learning process of resident physicians undergoing specialized medical training. Residents practice as physicians but are also engaged in continued clinical training toward specialist competence, thus representing the next generation of specialists. They represent several medical fields, work across departments and clinics, and participate in both traditional classroom-based learning and learning in clinical practice. In all, this makes this group relevant for the study of digital learning in various stages of medical education [*cf.* 21]. The overall aim of the study is to gain a comprehensive understanding of resident physicians’ experiences and perspectives concerning digital learning. The study is guided by the following research question: *How do resident physicians perceive and approach pedagogical opportunities and challenges in digital learning environments within the context of their medical training?*

## Related research

A previous review of the literature identified pedagogical factors (e.g. learner-instructor interaction), organizational factors (e.g. infrastructure, training), and technological factors (e.g. hardware and software) as influential for digital learning as part of residency education [20]. Individual digital skills, self-motivation, interactive elements, and relevance to practice are considered facilitating factors for creating a meaningful learning experience in digital learning environments in healthcare [22,23]. Education management and administration related to course planning, activities, and training in the systems being used are also necessary, along with technological aspects regarding reliability, usability, and accessibility related to the learning management systems per se [12,13].

As digital learning transitioned from being a minor part of the health professions education to becoming full-scale during the pandemic, medical residents were provided a wide range of learning opportunities, such as lectures, seminars, case discussions, and group discussions [6,7]. Studies focusing on the transition to digital learning in residency education during the pandemic showed a high level of satisfaction with digital learning activities among participants, but it was also evident that in-person training remains necessary since specific hands-on skills (e.g. ultrasound scanning, certain surgical skills, physical examinations, and emergency procedures) cannot be learned digitally [10,21].

In addition to replacing – and potentially enhancing or transforming – traditional ‘classroom’ activities such as lectures, seminars, and journal clubs, digital learning can support collegial communication, collaboration, knowledge sharing, and keeping up-to-date, which are essential aspects of learning and training, especially for specialized professions committed to lifelong learning, such as physicians [21]. Prior studies have highlighted the importance of social learning aspects that occur spontaneously in digital learning environments. For example, Joynes et al. (2017) studied the integration of technology into continuous professional development in healthcare and found that informal learning arose from ‘opportunistic encounters’ and more planned activities. Through co-designing content, digital learning environments can facilitate knowledge sharing and engagement in community building for self-sustainability and trustful sharing of domain-specific information through networking and collaboration over time [24]. While healthcare is a common site for research on workplace learning, studies focusing on informal digital learning are still lacking [25], and questions of the value and potential loss of social interactions that occur in medical school classrooms and workplace-based learning when shifting to digital learning remains [9,10].

## Theoretical background

The purpose of the previous section was to review related research on digital learning in postgraduate medical education and position this study in the previous literature. This section outlines the theoretical perspectives that have guided the study, to establish an understanding of the field of digital learning. Theoretical concepts in the intersection of healthcare and education serve as a framework for understanding the integration of technology and learning in healthcare, and the paper adopts a sociotechnical approach, where both social and technical aspects are accounted for.

Digital learning is not a new phenomenon; universities and other educational institutions have provided online and blended learning for decades, and there exists a body of research on the role of computers in education and how learning can be supported by digital technologies framed from various perspectives [26,27]. Research in CSCL (computer-supported collaborative learning) has focused explicitly on the actual use of technology for collaboration and problem-based learning, i.e. with an interest in understanding the process of learning (how) rather than the outcome (what) from a participant’s viewpoint [28]. Still, it remains a challenge to understand the relationship between digital technology, work, and learning and the implications of digital learning for continuous training and learning at work [29], especially in complex settings like healthcare, where there are high demands on the medical professionals to keep their skills and knowledge updated to solve problems in daily clinical practice [30,31].

When integrating technology into education, the Substitution, Augmentation, Modification, Redefinition (SAMR) model [32,33] provides a framework to conceptualize how teaching and learning change when new technology is introduced and the level of that change. The model is considered suitable for understanding pedagogical change (or possible transformation) driven by learning technologies in health professions education [34]. It can help analyze how the technology is being used according to different stages of technological involvement, from using digital technology to enhancing learning to transforming it. *Substitution* in digital learning can, for example, involve transferring and reusing traditional classroom lectures online with the same content and structure. *Augmentation* can be achieved by adapting the lecture to the digital format, breaking it into shorter segments, and making the content more focused. *Modification* might include publishing related content and materials, such as embedded video clips. *Redefinition* refers to a complete change that leads to improvements, which could mean presenting the lecture in a different context or using a ‘flipped classroom’ approach with group discussions either in person or in breakout rooms. While SAMR offers a structured way to reflect on the implementation of digital elements in teaching and learning practices, it has been critiqued for being too general and not considering specific contexts, for focusing on technology use rather than the learning process itself, and for its hierarchical structure, which can mislead educators to believe that only transformative uses of technology are valuable, overlooking the benefits of replacement and augmentation [32,34]. The PICRAT model [35] is another technology integration framework that combines Replacement, Amplification, Transformation [RAT; 36], a simplified version of SAMR, to describe the use of technology for teaching with PIC, which stands for Passive, Interactive or Creative and describes a learner’s relationship to technology [10]. While these models can be useful tools, they should be applied critically and in conjunction with other frameworks and considerations that acknowledge the importance of context and understanding of the learning process.

Digital learning can be described as an education strategy in which hardware and software technology facilitates learning by providing learning independent of time and distance [21,37]. Many countries, organizations, and education institutes worldwide have adopted digital learning to increase the chances that learners get higher quality learning [38]. However, this relationship is not a given. Traditionally, much research has studied digital learning from either the technological or the social aspects separately. This paper adopts the perspective that digital learning needs to be treated as a sociotechnical phenomenon because it consists of the social part (learners and instructors) as well as the technological part (the software and the hardware of the system), where both the social and technical aspects need to be taken into consideration together [29,39].

Pedagogical research shows that health professions education based on modern principles of adult learning, such as the importance of relevance, reflection, and active participation in learning, has greater potential to change the clinical practice of doctors than traditional lectures do [40,41]. This aligns with a sociotechnical approach to digital learning, which highlights the importance of focusing on the social situations and interactions, as well as cognitive aspects (e.g. ways of thinking and behaving) to foster an environment for digital learning that engage and motivate the students [29,39]. A sociotechnical approach is considered essential to developing a better understanding of why and for what purposes digital tools and educational methods are used and how they can contribute to lifelong learning, with a focus on the interplay between technology change and teaching methods [29,39,42].

## Methods

The methodological approach is qualitative, aiming to capture and understand participants’ experiences and views of digital learning during the pandemic and beyond. Semi-structured interviews with resident physicians were conducted to follow up, validate, and gain a more in-depth understanding of preliminary findings from previous analyses of course evaluation surveys [20].

### Setting and participants

The research setting is in Swedish public healthcare, at one of the larger non-university hospital groups, where around 200 doctors are enlisted in the hospital-wide residency educational program. In Sweden, medical residency is under the purview of public health authorities [43]. Hospital residency program directors at each hospital arrange mandatory courses, including specialty-specific subjects and general topics required by all specialties, such as leadership, science, communication, ethics, and law. In the case reported in this paper, the hospital group offers these courses twice a year. Before the pandemic, they were arranged as onsite courses, where the course participants and teachers met face-to-face, but during the pandemic, the organizers decided to teach these courses online.

All courses were evaluated with preexisting course evaluation questionnaires, comprising both quantitative measurements and qualitative assessment questions. The open-ended questions focused on participants’ experiences of participating digitally in the courses, as well as the strengths and weaknesses of the digital courses. They also included an opportunity to provide suggestions for improvement in the final question. All the physicians participating in the study were engaged in continuous training toward specialist competence as part of their medical training.

### Data collection

A qualitative approach was used to analyze answers to open-ended questions in course evaluation surveys (see appendix A, Supplementary Materials) for four of the courses held online during 2020–2021 (79 individuals, 119 participants, 138 answers in total) and semi-structured interviews with nine physicians from a cohort of participants enrolled in two or more digital courses. Course participants who attended at least two residency courses during the first year of the pandemic were invited to participate in an interview about their experiences participating in a digital course.

Participants received written and oral information about the study. This included consent for participation as well as consent for the publication of anonymized quotes in scientific journals. Nine physicians were included in the study after giving informed consent. Three were female, and six were male, representing different specialties. The interviews lasted between 20 and 50 min, were based on a semi-structured interview guide (see appendix B, Supplementary Materials), and were recorded and transcribed verbatim. No new concepts or additional themes were identified in the last interviews, and the research group then concluded that data saturation had been achieved.

### Analysis

The empirical data from interviews and course evaluations were analyzed collaboratively and iteratively through thematic analysis [44]. An initial coding scheme was developed inductively (data-driven) based on the research question and analysis of course evaluations to identify patterns, connections, and relationships in the material. Participants’ responses were condensed into shorter, simpler sentences, and relevant themes were identified from these responses. Microsoft Excel was used to organize these steps by creating tables to document the answers, codes, and themes. Table 1 provides an example of the initial coding process for the qualitative data analysis.

**Table 1.** Example of the coding process.

Question	Coding (condensed responses)	Theme
Specific suggestions on how the course can be carried out digitally?	Two moderators for better lecture management	Course management
	Instructors should have needed technology equipment and skills	Technology-related issue
	More digital activities to start discussions	Pedagogy-related issue

The next step included multiple readings and analyses independently by three of the four researchers in the author team (HVH, CMÖ, PP) to identify, distinguish, and analyze occurrences in the empirical material describing or referring to the digital transformation of learning (theoretically informed). The final step was to collaboratively sum up the findings of each theme, sort them into subthemes and main themes, and refine them to reach a final consensus. Initial coding was conducted manually, and the final analysis was supported by the qualitative data analysis software NVivo 12 [45]. After the initial coding, interview transcripts were formatted and imported into NVivo. They were then assigned to existing themes, followed by multiple coding to different nodes and re-coding nodes into broader or narrower themes during the final analysis, reflecting the iterative nature of the qualitative research process [46]. Table 2 provides a summary of the final main themes with example quotes from interviews and evaluations.

**Table 2.** Summary of themes with example quotes from interviews and evaluations.

Theme	Example quote
*Theme 1: Sociotechnical aspects* Relates to technology use during digital courses and perceived implications for learning	*However, it’s very varied how the course organizers manage it technically to keep it running smoothly throughout the day without interruptions […] It feels like the format tends to become more cathedral-like lectures again when it becomes online courses because it is easier technically, too, with less risk of interruptions and problems.* (R2) *There was a little problem with sound and such, but it was generally good.* (C1:26) *I find it easier to concentrate on the content when I’m there in person. I think it’s quite easy to zone out* (R8)
*Theme 2: Educational aspects* Relates to course design, content, pedagogical methods, and strategies in digital courses	*‘Group discussions worked really well, in my opinion. Then, you had to ask the questions in the chat and get answers from one of the course leaders while the lecture was going on, which made it possible to ask so many more questions than you would have been able to do.’* (R4) *‘…digital coffee rooms. That was very unexpected and nice!’* (C1:12) *I was quite surprised at how it almost became more effective this way. What I miss, though, during the actual lectures is that the questions became a bit more forced; it’s not as easy to just spontaneously ask questions* (R5)
*Theme 3: Administrative aspects* Relates to supporting structures and prerequisites for digital learning, such as course management, planning and access.	***‘****They had created websites where we had our schedules, as well as the course structure, and such things were on two websites, which was great.’* (R1) *‘The discussions on the articles would probably have worked much better if we had gotten them earlier.’* (C2:4) *There have been websites with educational materials, and they have also worked well. It has become easily accessible, and it has worked well for downloading documents and so on.* (R6)

Abbreviations in quotes: R = Respondent 1–9. C = Course 1–4: participant answer 1–138.

## Findings

Through our analysis of the course evaluations and the semi-structured interviews, we identified three main themes: *sociotechnical*, *educational, and administrative aspects of digital learning*. For a list of themes and example quotes, see Table 2. Each theme is presented and further discussed below, illustrated with sample quotes from the interviews and open-ended questions.

### Theme 1: Sociotechnical aspects of digital learning

The findings indicate that the physicians were satisfied with the digital courses. While a few participants commented that they at times struggled with the internet connection, referring to specific functions such as playing sound and video, in general, they did not experience any significant technical problems: ‘*Technically, I thought it worked quite well’* (R:9). Most of them had little or no experience of digital learning prior to the pandemic but commonly viewed themselves as digitally competent:’ *For us who are a little more computer-savvy, it’s a little easier, because I rarely think there have been any major problems’* (R6). Taking part in the courses remotely and using digital technology for learning was, thus, not particularly problematic. However, there were examples of unintended consequences, where digital features designed to support interaction and learning instead became a disturbing effect, as reflected in the following comment regarding the hand-raising function: *‘…but it didn’t work for the lecturer didn’t see it, so then it became that people just said, ‘excuse me, I have a question’ [so] we abandoned it*’ (R1). Likewise, with the use of Mentimeter (an audience engagement platform): ‘*It didn’t work that well, I must say, it becomes a little anonymous so you don’t see who it is from and then you can also ignore answering. So some answered very well, but others…’* (R1).

It was also perceived as a disruption if the lecturer was unprepared and stressed due to technical issues, as it interfered with the subject being taught. In those situations, one of the participants with experience or better digital skills often stepped in as a support function to solve the problem at hand. When commenting on technical issues related to the lectures, it was more directed towards the role of the teachers in terms of functioning technology, as well as digital skills, such as that the lecturer should have a reliable internet connection, a functional microphone, and be familiar with the technology: ‘*it can be lecturers sitting in different places, and where they realize that one of them hadn’t downloaded Teams […] or that person does not have access rights […] so there have been some situations when you think that yes this could you have fixed better but where they haven’t anticipated those problems’* (R2).

Several participants emphasized the importance of having working webcams during the lectures to facilitate communication between participants, especially during discussions, and to keep instructors enthusiastic: ‘*…to see all participants, and maybe this would encourage people to talk more rather than typing in the chat’* (C1:3). On the other hand, participants also expressed positive experiences of using the chat for questions to structure the discussion instead of everyone talking aloud in the Zoom room all at once. When it comes to specific software used during the courses, participants emphasized that they wanted to use the same platform and not have to spend time and effort learning how to navigate a new platform in the middle of the course: *‘Zoom has usually worked better…Skype has messed up in several other courses as well’* (C2:4). Some of the course elements that could be improved, regarding the technical aspects, relate to the way lecture materials were presented online as participants struggled with seeing material presented during online lectures: ‘*squeezed a lot of text into small boxes by changing the size’* (C1:18).

### Theme 2: Educational aspects of digital learning

It was evident that the participants valued interactive learning elements and teamwork, and they also emphasized the importance of the social and informal aspects of the courses. As one respondent commented, it is a way of getting acquainted and learning more about each other through interacting and talking during the courses, which, in turn, improves the discussions and learning. ‘*The drawbacks are that you don’t know people, so it’s harder to start a conversation with someone […], so it takes a while before it becomes good flow’* (R9). The importance of knowing other participants was considered a factor that facilitates digital learning: *‘…since I was already familiar with almost everyone, it worked very well. I think it is more difficult if you do not know anyone already’* (C1:12). Several participants commented that they appreciated the possibility of staying online and talking after and between lectures, both with each other and asking follow-up questions to course leaders. Social elements online were also appreciated: *‘Or you might have a coffee chat or such things, so that you sit and talk, that you don’t just turn off in between, I don’t know’* (R5).

Moreover, the respondents reflected specifically on the advantages and disadvantages of online versus face-to-face courses when integrating interactive activities during the courses. On the one hand, participating online was perceived as inhibiting for the discussions, especially in the larger group: *‘It certainly won’t be the same when the lecturer asks a question to a whole group, to get some kind of discussion going, it just doesn’t work in digital form’* (C3:4). On the other hand, discussions in breakout rooms were highly appreciated and highlighted as a strength of the digital courses, both in the evaluations and interviews. The positive aspects of online discussions for group dynamics, learning, and reflection are illustrated by the following quote: *‘It was incredibly effective, quicker to get started and talk about what you would do in these digital spaces than in real life. There were also very good dynamics when changing constellations from time to time. It was easy to ensure that everyone was heard, actually easier digitally than in real life’* (C1:18).

Finally, related to the courses’ pedagogical content, many physicians highlighted that case-based discussion, in particular, facilitated learning and reflection: ‘*We had a clear question we were supposed to discuss […] it became a little easier for everyone to talk and raise a little, that it became a better discussion’* (R8). Regardless of whether they took place online or face-to-face, it was perceived as leading to a deeper understanding of the subject and, overall, a better learning experience: *‘The discussions around lectures and patient case presentations were enriching […] a very good tool to be able to quickly answer the difficult questions in a structured and objective way in practice*’ (C4:16). Moreover, digital features such as polls, quizzes, and Mentimeter were appreciated and mentioned as examples of when digital technology could enhance the learning experience to avoid zoning out and losing concentration. Still, when it comes to more soft values, such as around supervision and consultations where it’s a lot about reasoning, the digital format was perceived as challenging for the learning: *‘…because it’s more discussions and maybe you can lecture a little, but it’s a different way of learning, and then it was challenging with the digital platform’* (R1).

### Theme 3: Administrative aspects of digital learning

The physicians shared positive experiences of participating in digital courses during the pandemic. Many of them wanted to first and foremost express that they were satisfied with the digital format, especially as the other option would have been to cancel: *‘…I genuinely did not expect it, but, I must say, it worked out surprisingly well after all’* (R8). Nonetheless, after highlighting the positive aspects, they also commented on issues that had not worked out and suggested improvements. It was apparent that information, structure, and logistics around the course days became even more critical when the courses were provided in digital form.

Practical issues highlighted in course evaluations and interviews are concerned, to a large extent, with time management, planning, and preparations. Participants noted that lecturers often skipped slides at the end of the presentation, highlighting the importance of time management and preparation: *‘I think the lecturers’ perception of time is much more difficult too when they are online*’ (R2). Partly due to technical issues with screen sharing and a longer time to connect and get started: ‘*Screen sharing and such, that’s also a threshold, that people should know now how to show the slides, but it’s just user experience*’ (R6). But also related to content and presentation technique, where respondents reflected on the need for digital pedagogy and adapting the lectures to the digital format rather than simply copying the traditional lecture style: *‘People should have better PowerPoint skills and also some more pedagogical tricks, that you can’t fill a whole page with text, and of course, it’s more about pedagogy and how they are trained in it than this e-learning part, but it becomes such an important part of it’* (R6).

Participating in lectures online was perceived as more demanding. Participants suggested adjustments to the design of the course, for example, incorporating short breaks and more interactive elements in the afternoons, such as pop-up questions, to create engagement, especially during long lectures, as it is easy to zoom out and lose concentration: *‘one-hour presentation is a little too much, especially on web-based courses where you can easily lose focus. I would have liked a little more leg stretching’* (C4:14).

The distribution of course material beforehand and the organizing of breakout rooms were other practical concerns raised by participants, for example, to think through the size of the groups not to be too many but also not too few: *‘At one point we were only two in a group, but on the other hand, it felt rather as if we were too many at the group exercise the other day’* (C1:5). Another element mentioned by participants as facilitating was using the chat during lectures to write comments or ask questions without interrupting the instructor: ‘*That you are a little clearer about what applies, like that everyone should have video on, or that all questions are written in the chat. A little clearer rules of order if you want to call it that’* (R6). Having a moderator or assigning someone responsible for the digital, such as monitoring the chat and distributing the word among the participants, was perceived as facilitating: ‘*Like a moderator and kept a little track of times and a little track of people who raised their hand and so on, it was very professional’* (R6).

## Discussion

In this paper, we have addressed key elements that may enhance or constrain digital learning for physicians in residency programs—focusing on sociotechnical, educational, and administrative aspects—and corresponding digital learning strategies arising from using digital learning environments. We contribute to research on the interplay between digital technology and learning in health profession education. We also respond to calls for more research and empirical insights into the effects of digital learning during the pandemic and beyond [21,34,47].

First, we uncovered physicians’ views, concerns, and ideas related to software technology, including the digital platform used in the lectures and other digital tools and equipment used during their participation in the online courses. Our research findings support previous studies that emphasize the pandemic as a catalyst for digital learning [7,9], acknowledging challenges related to the ‘digital divide’ between educators and learners [48] and the potential of millennial residents’ familiarity with social networks and digital technology [49]. Strategies undertaken by the physicians as they interact with both people and technologies in new digital learning environments include using the private channel in the group chat or joining with or without the camera, for example, when switching between breakout sessions and lectures. Challenges initially described as technological, often turned out to be more of social character, for example related to support for digital learning technique or not lack of understanding of the purpose or usefulness of using specific features and tools. In all, this illustrates the importance of a sociotechnical approach and understanding digital learning in context [18,32,34] while not black-boxing the technology per se [24,30].

Secondly, our findings reveal insights into physicians’ perceptions of digital learning related to educational issues, highlighting the important role interactive communication and group discussions have for their learning. The transition from traditional learning (onsite, face-to-face) to digital learning (online, remotely) was perceived as sudden, urgent, and unplanned [50,51], but it also led to positive effects in terms of innovative and creative solutions. Participants reported on the benefits of digital learning mainly as replacement and enhancement of the learning experience, e.g. group discussions in breakout rooms and using polls and Mentimeter to support interactivity during lectures, which were considered elements that added value [*cf.* 33, 35]. This suggests that more focus should be placed on supporting how people learn as they interact and engage with each other and the technology in novel ways, especially during prolonged crises [8,21,47]. Resident physicians can benefit from digital learning because of the nature of their work, with varied work shift schedules that may prevent them from attending traditional education lectures [18,21]. In this regard, digital learning can potentially transform residency education if this mode of learning is maintained post-pandemic.

Finally, the participants’ reflections on administrative issues related to course design and lecture management illustrate different structure and informational support needs between digital learning and traditional courses. As the use of digital technology for learning purposes continues to grow, it’s important to develop strategies for better understanding how digital tools and educational methods can be integrated to support the need for educational IT, digital skills, and relevant support and training [23]. Simultaneously, teaching in Zoom and managing the chat can be challenging [10], as illustrated in our findings by the fact that participants sometimes stepped in to help moderate group chats and question sessions. Loss of social interactions and engagement have been identified as common barriers to digital learning, and our findings reflect common strategies for maintaining interactivity and engagement in digital learning environments, such as virtual hand raising, screen sharing, built-in chat functions, polls, and breakout sessions [9,10,21]. An interesting finding from this study is, therefore, the importance of social and informal networking and relationships built up *during the courses*, suggesting that this ‘hidden curriculum’ is an equally important part of the learning that can be both enabled and constrained in digital learning [19,24,52].

To sum up, our findings show how resident physicians perceive pedagogical opportunities and challenges in digital learning environments during their medical training, highlighting the importance of a holistic approach that considers all three perspectives when incorporating learning technology in education and training. Aligning with the PICRAT model [34], our study illustrates how respondents experienced and appreciated the interactive and creative use of technology as part of digital learning. The digital courses included elements of *replacement*, such as the reuse of lectures, but also a wide variety of *amplification*, such as digital polls, breakout rooms and digital coffee breaks. As can be expected, since all courses in this study had been redesigned from previous onsite courses, without expertise in digital pedagogy, we did not find examples of *transformation* in terms of a complete change of the pedagogical approach. For future courses, we believe that competence in digital pedagogy (i.e. both technological and pedagogical competence) early in the course design is crucial for pedagogical transformation in medical education.

Based on our empirical insights and discussion of the findings, we suggest the following considerations to enhance medical education and training through pedagogical transformation:

- *Considerations for sociotechnical learning approaches:* The study emphasizes the importance of a sociotechnical approach to digital learning that considers learning through interactions with others and with technology, along with relevance to practice, to facilitate learning in medical education. Our findings are supported by previous research, for example, physicians emphasizing the importance of well-integrated, user-friendly digital technology as a prerequisite for learning [53] while also considering the significance of digital literacy among educators as equally important [54].
- *Educational considerations for digital learning:* The importance of recognizing learning as a social and cognitive process is reflected in this study through the value of interactive communication and networking during digital courses, emphasizing its role in the ‘hidden curriculum’ of learning. This insight strengthens that fostering social connections is crucial in digital learning environments [55,56]. Digital learning was initially perceived as a barrier to interactive communication and dialogue but was, over time, enhanced by incorporating digital features to facilitate online communication and group discussions, including social and informal interactions, which were also maintained post-pandemic.
- *Administrative considerations for digital courses:* Clearly, digital learning requires structure and attention to course administrative issues. The study emphasizes the importance of integrating digital tools and educational methods and providing informational support that can help understand the usefulness and relevance of digital learning, as this aspect has been described as influential for the willingness to embrace and use digital learning tools in previous research [57]. Although digital learning initially was perceived with some resistance, it was in hindsight seen as a positive effect, as participants adjusted to the new ways of learning and discovered tools that facilitate time management, organization of course material, and studying.

The strengths of this qualitative study, using multiple sources (course evaluations and interviews), include credibility through empirical insights into resident physicians’ authentic experiences and perceptions of digital learning, identification of key aspects that may enhance or constrain the learning, and strategies used by the physicians to address these [46]. Our findings highlight both the advantages and disadvantages of digital learning from the perspective of physicians, suggesting that a combination of synchronous and asynchronous learning approaches could be considered in the design of future residency education. Theoretically, the study contributes to a better understanding of how digital learning environments, including virtual lectures and seminars, can be designed and incorporated into residency education and health professions education more broadly. The study limitations include the use of preexisting course evaluation questionnaires, not specifically designed or validated for this research. Additionally, the results are limited to the context of one Swedish hospital, so they may not apply or be relevant to other settings, and there is always a risk that the participants who volunteered for the study may have a stronger interest in digital learning compared to the general physician population. Still, the qualitative and reflexive approach has enabled continuous data analysis and refining of the research questions and themes and the study provides a detailed description of the context and the characteristics of the participants to enable the reader to assess transferability. A clearly outlined methodology including transparency in the data collection process, data-driven coding, and iterative thematic analysis ensures the dependability of the study [46].

## Conclusion

Through a qualitative approach, based on thematic analysis of course evaluations and semi-structured interviews, findings from this study highlight sociotechnical, educational, and administrative aspects as influential for digital learning in residency education. It illustrates how digital learning can be perceived as simultaneously constraining and enabling, and it discusses strategies used by physicians to address these challenges from all three perspectives. The study confirms and extends prior studies on digital learning in healthcare, strengthening the need for holistic approaches and illustrating how the (forced) shift to digital learning can create innovative thinking and learning opportunities. Implications for practice include better knowledge and understanding of how a digital learning environment, in general, and learning through virtual lectures and seminars can be designed and incorporated into health professions education to increase their usefulness.

## Supplementary Material

Supplemental Material

## Data Availability

We have full control of all primary data, and we agree to allow the journal to review our data if requested. The datasets used and/or analyzed during the current study are available from the corresponding author in response to reasonable requests.

## References

[1] Konstantinidis ST, Bamidis PD, Zary N. Digital innovations in healthcare education and training. [Internet]. London: Academic Press; 2021; [cited 2024 Oct 10]. Available from: https://research.ebsco.com/linkprocessor/plink?id=833726c9-eda1-3cf5-8ede-d825b103632b

[2] Bates AW. Teaching in a digital age. Vancouver BC Tony Bates Associates Ltd.; 2015.

[3] Cohen A, Nørgård RT, Mor Y. Hybrid learning spaces – design, data, didactics. Brit J Educat Tech. 2020;51(4):1039–1044. doi: 10.1111/bjet.12964.

[4] Wheeler S. e-Learning and digital learning. In Seel NM, editor. Encyclopedia of the sciences of learning. Boston, MA: Springer US; 2012. p. 1109–1111.

[5] Gegenfurtner A, Ebner C. Webinars in higher education and professional training: a meta-analysis and systematic review of randomized controlled trials. Educat Res Rev. 2019;28:100293. doi: 10.1016/j.edurev.2019.100293.

[6] Dedeilia A, Sotiropoulos MG, Hanrahan JG, et al. Medical and surgical education challenges and innovations in the COVID-19 era: a systematic review. In Vivo. 2020;34(3 Suppl):1603–1611. doi: 10.21873/invivo.11950. 32503818 PMC8378024

[7] Reyna J. Twelve Tips for COVID-19 friendly learning design in medical education. MedEdPublish. 2020;9:103. doi: 10.15694/mep.2020.000103.1. 38090049 PMC10712607

[8] Hodges CB, Moore S, Lockee BB, et al. The difference between emergency remote teaching and online learning; 2020. EduCAUSE Review [Internet]. Available from: https://er.educause.edu/articles/2020/3/the-difference-between-emergency-remote-teaching-and-online-learning.

[9] Woolliscroft JO. Innovation in response to the COVID-19 pandemic crisis. Acad Med. 2020;95(8):1140–1142. doi: 10.1097/ACM.0000000000003402. 32282372 PMC7188042

[10] Khamees D, Peterson W, Patricio M, et al. Remote learning developments in postgraduate medical education in response to the COVID-19 pandemic – a BEME systematic review: BEME Guide No. 71. Med Teach. 2022;44(5):466–485. doi: 10.1080/0142159X.2022.2040732. 35289242

[11] Chikurteva A, Spasova N, Chikurtev D, editors. E-learning: technologies, application and challenges. 2020 XXIX International Scientific Conference Electronics (ET); 2020; IEEE. doi: 10.1109/ET50336.2020.9238176.

[12] Ananga P. Pedagogical considerations of E-learning in education for development in the face of COVID-19. IJTES. 2020;4(4):310–321. doi: 10.46328/ijtes.v4i4.123.

[13] Vaona A, Banzi R, Kwag KH, et al. E-learning for health professionals. Cochrane Database Syst Rev. 2018;1(1):Cd011736. doi: 10.1002/14651858.CD011736.pub2. 29355907 PMC6491176

[14] Fosslien L, Duffy MW. How to combat zoom fatigue. Harv Bus Rev. 2020;29:1–6.

[15] Daniel M, Gordon M, Patricio M, et al. An update on developments in medical education in response to the COVID-19 pandemic: a BEME scoping review: BEME Guide No. 64. Med Teach. 2021;43(3):253–271. doi: 10.1080/0142159X.2020.1864310. 33496628

[16] Gordon M, Patricio M, Horne L, et al. Developments in medical education in response to the COVID-19 pandemic: a rapid BEME systematic review: BEME Guide No. 63. Med Teach. 2020;42(11):1202–1215. doi: 10.1080/0142159X.2020.1807484. 32847456

[17] Grafton-Clarke C, Uraiby H, Gordon M, et al. Pivot to online learning for adapting or continuing workplace-based clinical learning in medical education following the COVID-19 pandemic: a BEME systematic review: BEME Guide No. 70. Med Teach. 2022;44(3):227–243. doi: 10.1080/0142159X.2021.1992372. 34689692

[18] Wittich CM, Agrawal A, Cook DA, et al. E-learning in graduate medical education: survey of residency program directors. BMC Med Educ. 2017;17(1):114. doi: 10.1186/s12909-017-0953-9. 28697744 PMC5504987

[19] Vallo Hult H, Master Östlund C, Pålsson P, et al. Designing for digital transformation of residency education – a post-pandemic pedagogical response. BMC Med Educ. 2023;23(1):421. doi: 10.1186/s12909-023-04390-2. 37291569 PMC10248334

[20] Abovarda A, Vallo Hult H, Master Östlund C, Pålsson P, editors. E-learning as Part of Residency Education. 33rd Medical Informatics Europe Conference, MIE2023, was held in Gothenburg, Sweden; 2023, from 22 to 25 May 2023; IOS Press.

[21] Hopcan S, Polat E, Albayrak E. Research trends in e-learning practices for postgraduate medical education: a systematic review. Educ Inf Technol. 2024;29(5):5921–5945. doi: 10.1007/s10639-023-12035-6.

[22] Tsodikova O, Korzh O, Hyria M. Information and communication technologies in postgraduate training of primary care doctors: a new look at the problem of using online resources during the Covid-19 pandemic. TSSJ. 2020;8:60–63. doi: 10.47577/tssj.v8i1.661.

[23] Vallo Hult H, Hansson A, Gellerstedt M. Digitalization and physician learning: individual practice, organizational context, and social norm. J Contin Educ Health Prof. 2020;40(4):220–227. doi: 10.1097/CEH.0000000000000303. 33284172 PMC7707155

[24] Vallo Hult H, Islind AS, Master Östlund C, et al. Sociotechnical Co-design with General Pediatricians: ripple Effects through Collaboration in Action. Salt Lake City, UT: AMCIS; 2020.

[25] Curran V, Fleet L, Simmons K, et al. Adoption and use of mobile learning in continuing professional development by health and human services professionals. J Contin Educ Health Prof. 2019;39(2):76–85. doi: 10.1097/CEH.0000000000000243. 30908401

[26] Östlund C. Design for e-training [Doctoral thesis, monograph]. Köpenhamn Copenhagen Business School Press; 2017.

[27] Hiltz SR. The virtual classroom: learning without limits via computer networks. Norwood, N.J.: Ablex Pub. Corp; 1994.

[28] Koschmann TD. CSCL: theory and practice of an emerging paradigm. Hillsdale, NJ: Lawrence Erlbaum; 1996.

[29] Islind AS, Norström L, Vallo Hult H, et al. Socio-technical interplay in a two-sided market: the case of learning platforms. In: Metallo, C., Ferrara, M., Lazazzara, A., Za, S. editors. Digital Transformation and Human Behavior. Lecture Notes in Information Systems and Organisation, vol 37. Springer, Cham. 2021.

[30] Vallo Hult H, Master Östlund C. Towards design principles for the three phases of physicians’ information seeking activities. In: Chandra Kruse L, Seidel S, Hausvik GIm, editors.The next wave of sociotechnical design. Cham: Springer International Publishing; 2021. p. 65–70.

[31] Daei A, Soleymani MR, Ashrafi-Rizi H, et al. Clinical information seeking behavior of physicians: a systematic review. Int J Med Inform. 2020;139:104144. doi: 10.1016/j.ijmedinf.2020.104144. 32334400

[32] Hamilton ER, Rosenberg JM, Akcaoglu M. The Substitution Augmentation Modification Redefinition (SAMR) Model: a critical review and suggestions for its use. TechTrends. 2016;60(5):433–441. doi: 10.1007/s11528-016-0091-y.

[33] Puentedura R. Transformation, technology, and education. 2006; [cited 2024 Oct 10]. Available from: http://hippasus.com/resources/tte/Grainger R.

[34] Grainger R, Liu Q, Gladman T. Learning technology in health professions education: realising an (un) imagined future. Med Educ. 2024;58(1):36–46. doi: 10.1111/medu.15185. 37555302

[35] Kimmons R, Graham CR, West RE. The PICRAT model for technology integration in teacher preparation. Contemporary Issues Technol Teacher Educat. 2020;20(1):176–198.

[36] Hughes J, Thomas R, Scharber C, editors. Society for information technology & teacher education international conference; 2006: Association for the Advancement of Computing in Education (AACE) Assessing technology integration: the RAT–replacement, amplification, and transformation-framework.

[37] Alhabeeb A, Rowley J. E-learning critical success factors: comparing perspectives from academic staff and students. Computers Educat. 2018;127:1–12. doi: 10.1016/j.compedu.2018.08.007.

[38] Gapsalamov A, Bochkareva T, Vasilev V, et al. Comparative analysis of education quality and the level of competitiveness of leader countries under digitalization conditions. J Soc Stud Educat Res. 2020;11(2):133–150.

[39] Upadhyaya KT, Mallik D. E-learning as a socio-technical system: an insight into factors influencing its effectiveness. Business Perspect Res. 2013;2(1):1–12. doi: 10.1177/2278533720130101.

[40] Holmgren D, Skyvell-Nilsson M, Wekell P. Combining learning for educators and participants in a paediatric CPD programme. BMC Med Educ. 2019;19(1):28. doi: 10.1186/s12909-019-1461-x. 30665454 PMC6341706

[41] Davis N, Davis D, Bloch R. Continuing medical education: AMEE education guide No 35. Med Teach. 2008;30(7):652–666. doi: 10.1080/01421590802108323. 18777424

[42] Vallo Hult H, Byström K. Challenges to learning and leading the digital workplace. Stud Continu Educat. 2021;3(44):460–474.

[43] The Doctors’ Specialization Service The National Board of Health and Welfare’s constitution. ; 2015.

[44] Braun V, Clarke V. Using thematic analysis in psychology. Qualitat Res Psychol. 2006;3(2):77–101. doi: 10.1191/1478088706qp063oa.

[45] Bazeley P, Jackson K. Qualitative data analysis with NVivo. London: SAGE; 2013.

[46] Korstjens I, Moser A. Series: practical guidance to qualitative research. Part 4: trustworthiness and publishing. Eur J Gen Pract. 2018;24(1):120–124. doi: 10.1080/13814788.2017.1375092. 29202616 PMC8816392

[47] Frenk J, Chen LC, Chandran L, et al. Challenges and opportunities for educating health professionals after the COVID-19 pandemic. Lancet. 2022;400(10362):1539–1556. doi: 10.1016/S0140-6736(22)02092-X. 36522209 PMC9612849

[48] Teele SA, Sindelar A, Brown D, et al. Online education in a hurry: delivering pediatric graduate medical education during COVID-19. Prog Pediatr Cardiol. 2021;60:101320. doi: 10.1016/j.ppedcard.2020.101320. 33169056 PMC7609226

[49] Ashton E, Skayem C, Ouazana-Vedrines C, et al. Junior doctors: when fresh blood fast-tracks the fight against COVID-19. Postgrad Med J. 2021;97(1145):185–187. doi: 10.1136/postgradmedj-2020-138782. 32883766 PMC10016853

[50] Appolloni A, Colasanti N, Fantauzzi C, et al. Distance learning as a resilience strategy during Covid-19: an analysis of the Italian context. Sustainability. 2021;13(3):1388. doi: 10.3390/su13031388.

[51] Kumar P, Saxena C, Baber H. Learner-content interaction in e-learning-the moderating role of perceived harm of COVID-19 in assessing the satisfaction of learners. Smart Learn Environ. 2021;8(1):1–15. doi: 10.1186/s40561-021-00149-8. PMC8050475 40477398

[52] Holmgren D, Vallo Hult H, Wekell P. Integrating a pedagogic course in a CPD programme for paediatricians at out-patient clinics. J Eur CME. 2021;10(1):1862981. doi: 10.1080/21614083.2020.1862981. 33552677 PMC7850427

[53] Wang C-H, Lin H-CK. Constructing an affective tutoring system for designing course learning and evaluation. J Educat Comput Res. 2018;55(8):1111–1128. doi: 10.1177/0735633117699955.

[54] Notagar S. Digital design and its prospects in higher education. Int J Eng Technol Management Sci. 2023;7(5):67–73.

[55] Kim HW. Social cognitive theory and medical education: how social interactions can inform learning. Korean Med Educ Rev. 2020;22(2):67–76. doi: 10.17496/kmer.2020.22.2.67.

[56] Horsburgh J, Ippolito K. A skill to be worked at: using social learning theory to explore the process of learning from role models in clinical settings. BMC Med Educ. 2018;18(1):156. doi: 10.1186/s12909-018-1251-x. 29970052 PMC6029173

[57] O’Doherty D, Dromey M, Lougheed J, et al. Barriers and solutions to online learning in medical education – an integrative review. BMC Med Educ. 2018;18(1):130. doi: 10.1186/s12909-018-1240-0. 29880045 PMC5992716

