# Supplementary material for: Digital learning strategies in residency education
Source: Ann Med. 2024 Dec 18;57(1):2440630. doi: 10.1080/07853890.2024.2440630 (PMC11656749; doi:10.1080/07853890.2024.2440630)
Supplement: Supplemental Material [file IANN_A_2440630_SM4908.zip › Suppl_Mat/Appendix_A_Course_evaluation (1).docx]

**Appendix A: Course evaluation**

Questions asked in the course evaluations (translated from Swedish)

1. Please rate your overall experience of the course on a scale of 1 to 6 *(1=learned nothing at all, 6=very educational).*
2. How much do you agree with the statement "A digital course works just as well for learning as a physical course" on a scale of 1 to 6 *(1=Do not agree at all, 6=Fully agree)*?
3. What part of the course did you find most educational?
4. Was there any part of the course that was less significant or uninteresting?
5. Was there any part of the course that didn’t work well in a digital format?
6. Please provide suggestions for changes for next time.
7. Please provide concrete suggestions on how the course can be conducted better digitally
