# Supplementary material for: Digital learning strategies in residency education
Source: Ann Med. 2024 Dec 18;57(1):2440630. doi: 10.1080/07853890.2024.2440630 (PMC11656749; doi:10.1080/07853890.2024.2440630)
Supplement: Supplemental Material [file IANN_A_2440630_SM4908.zip › Suppl_Mat/Appendix_B_interview_guide (1).docx]

**Appendix B: Interview guide**

Questions that will be asked during the interview:

- Can you briefly describe what you work with and your experiences with distance learning before and during the pandemic
- What was it like to complete the course digitally?
- Were digital tools used during the course? (e.g., Zoom, Skype, Mentimeter, video, etc.)
- How was your experience with the use of technical solutions?
- What advantages and disadvantages do you see with the digital form compared to a face-to-face course?
- Are there course elements that work particularly well in a digital course? If so, what are they?
- Are there course elements that work particularly poorly in a digital course? If so, what are they?
- Is there something in the digital course design that you would like to change next time?
- What is your opinion about courses that blend digital and face-to-face course days?
- Is there anything we haven’t covered during the interview that you would like to add?
